# Hallmarks of the Mott-metal crossover in the hole-doped pseudospin-1/2 Mott insulator Sr_2_IrO_4_

**DOI:** 10.1038/ncomms11367

**Published:** 2016-04-22

**Authors:** Yue Cao, Qiang Wang, Justin A. Waugh, Theodore J. Reber, Haoxiang Li, Xiaoqing Zhou, Stephen Parham, S.-R. Park, Nicholas C. Plumb, Eli Rotenberg, Aaron Bostwick, Jonathan D. Denlinger, Tongfei Qi, Michael A. Hermele, Gang Cao, Daniel S. Dessau

**Affiliations:** 1 Department of Physics, University of Colorado, Boulder, Colorado 80309, USA; 2 Department of Physics, Incheon National University, Incheon 22012, Korea; 3 Swiss Light Source, Paul Scherrer Institut, Villigen PSI CH-5232, Switzerland; 4 Advanced Light Source, Lawrence Berkeley National Laboratory, Berkeley, California 94720, USA; 5 Department of Physics and Astronomy, Center for Advanced Materials, University of Kentucky, Lexington, Kentucky 40506, USA

## Abstract

The physics of doped Mott insulators remains controversial after decades of active research, hindered by the interplay among competing orders and fluctuations. It is thus highly desired to distinguish the intrinsic characters of the Mott-metal crossover from those of other origins. Here we investigate the evolution of electronic structure and dynamics of the hole-doped pseudospin-1/2 Mott insulator Sr_2_IrO_4_. The effective hole doping is achieved by replacing Ir with Rh atoms, with the chemical potential immediately jumping to or near the top of the lower Hubbard band. The doped iridates exhibit multiple iconic low-energy features previously observed in doped cuprates—pseudogaps, Fermi arcs and marginal-Fermi-liquid-like electronic scattering rates. We suggest these signatures are most likely an integral part of the material's proximity to the Mott state, rather than from many of the most claimed mechanisms, including preformed electron pairing, quantum criticality or density-wave formation.

## Abstract (web summary)

The physics of Mott insulators is obscured by the interplay between competing orders and fluctuations. Here, the authors track the evolution of the electronic structure of Mott insulator strontium iridate as the iridium atoms are replaced by rhodium, providing insight into this exotic state of matter.

## Graphical abstract

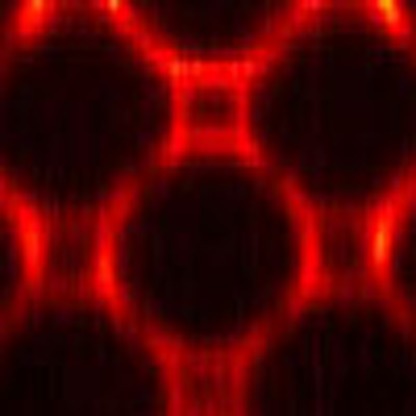

Finding universal features in interacting electronic systems is a major theme in modern condensed matter research. By universality we refer to the low-energy properties that do not depend on the details of the interactions. For materials with relatively weak electron correlations, the low-energy excitations are well-described by the Fermi liquid theory. For doped Mott insulators where correlations are strong, pinpointing the hallmarks common to all Mott-metal crossovers^1^,^2^,^3^ has proven a formidable task. This could largely be attributed to the long candidate list of competing electronic orders, including long-range magnetic order and Fermi surface instabilities, among others, which yield a complex global doping-temperature phase diagram. For example, while the past three decades have witnessed tremendous progress in characterizing the phenomenology of the high-Tc cuprates, the interpretation of most of these experimental observations remains highly controversial. It has become essentially inseparable whether such exotic phenomena as pseudogaps and marginal Fermi liquid (MFL) scattering rates^2^,^3^,^4^,^5^ arise from the metal-insulator transition, certain density-wave instabilities^6^ or are fluctuations of the superconductivity^7^. Moreover, the charge insulation in most known Mott insulators arises solely from the Coulomb repulsion. It would be highly desired to study new doped Mott insulators, especially those with a cleaner phase diagram (thus fewer competing orders) and a different mechanism that forbids electron double-occupancy.

Sr_2_IrO_4_ has attracted much interest recently as a new family of Mott insulator^8^,^9^, with the potential of realizing novel phases of matter^10^ and achieving higher superconducting T_C_^11^. The Ir-O planes are similar to the Cu-O planes in cuprates, with the Ir atoms sitting at the centre of the Ir-O octahedra, except for the 22° Ir-O-Ir bond angle (Fig. 1a). There are five t_2g_ electrons per Ir. The Ir t_2g_ level splits into the *J*_3/2_ doublet (filled with four electrons) and the *J*_1/2_ singlet as a result of the strong spin-orbit coupling. The half-filled *J*_1/2_ further splits into lower (filled with 1 electron) and upper (empty) bands, with this splitting general believed to be due to the Coulomb repulsion, which is why these bands are considered upper and lower Hubbard bands (UHB and LHB, see Fig. 1c)^9^. The insulating behaviour in Sr_2_IrO_4_ derives from the coupling of strong spin-orbit interaction with Coulomb repulsions, which is drastically different from that in cuprates. However, the Sr_2_IrO_4_ parent compound, just like undoped cuprates, is antiferromagnetically ordered^12^. Driving Sr_2_IrO_4_ towards metallicity thus provides a unique opportunity to investigate the universal features of the Mott-metal crossover.

Different approaches of doping Sr_2_IrO_4_ and related compounds have been found^13^,^14^,^15^,^16^,^17^, and of special interest is the Sr_2_Ir_1−*x*_Rh_*x*_O_4_ series. With as little as 4% Rh substitution, the normalized resistivity drops by 10^4^. The long-range magnetic order decays more slowly and still survives with a T_N_∼17 K for 15% Rh (see Supplementary Fig. 1). Rh is directly above Ir in the periodic table, so is expected to be isovalent. It has been proposed that the metallicity in the Rh-doped iridates comes from the reduced spin-orbit coupling of Rh (due to the smaller atomic number) which then leads to the reduced splitting of the *J*_3/2_ and *J*_1/2_ bands^15^,^16^, as well as the formation of in-gap states^16^ (Fig. 1e). As we will show below, the Rh atoms in fact act as effective hole dopants to Sr_2_IrO_4_ (Fig. 1d). The chemical potential moves to the top of the LHB, without major band renormalizations.

So far, no superconductivity has yet been reported in these Rh-doped compounds, which is different from doped cuprates. The absence of superconductivity reduces possible competing orders and makes the Rh-doped iridates a cleaner system to study—the long-range canted antiferromagnetism (AF) is the only confirmed order in the system. In the following, we show in the effectively hole-doped Sr_2_IrO_4_ the emergence of pseudogaps, Fermi arcs and marginal-Fermi-liquid-like electronic scattering rates, features first reported and/or most famous in doped cuprate Mott insulators. It appears these iridates resemble cuprates not only structurally but also in terms of electronic structures.

## Results

### Effective hole doping via Rh substitution

**Figure 1:**
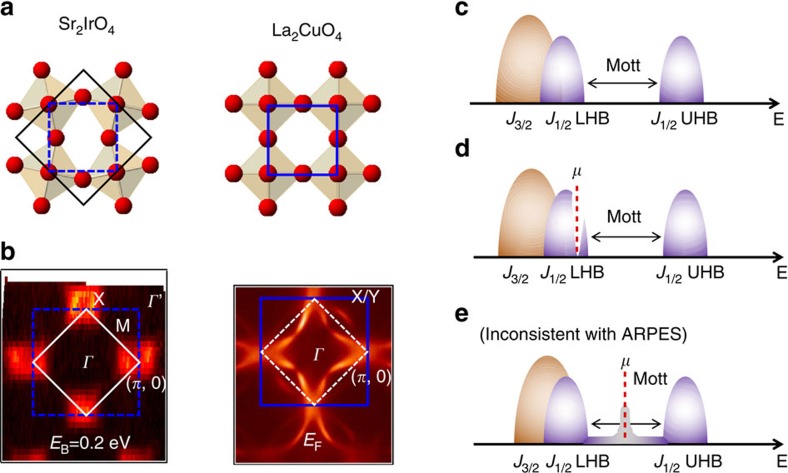
Sr_2_IrO_4_ as a Mott insulator on the square lattice. (**a**) The real-space unit cells of Sr_2_IrO_4_ and La_2_CuO_4_. (**b**) The k-space unit cells of the same, with matching colour scaling and with near-*E*_F_ ARPES spectral weight. Ignoring the 22° Ir-O twists gives the blue cells in real and k-space, and corresponds to the regular unit cell of La_2_CuO_4_. Including these twists in Sr_2_IrO_4_ (black, (**a**)) back-folds the k-space cell in k-space (white), similar to the AF order in the parent cuprates. (**c**) The formation of the Mott gap in Sr_2_IrO_4_ as a result of the spin-orbit coupling and Coulomb interaction. (**d**,**e**) Possible schematics of chemical potential evolution with Rh doping.

**Figure 2:**
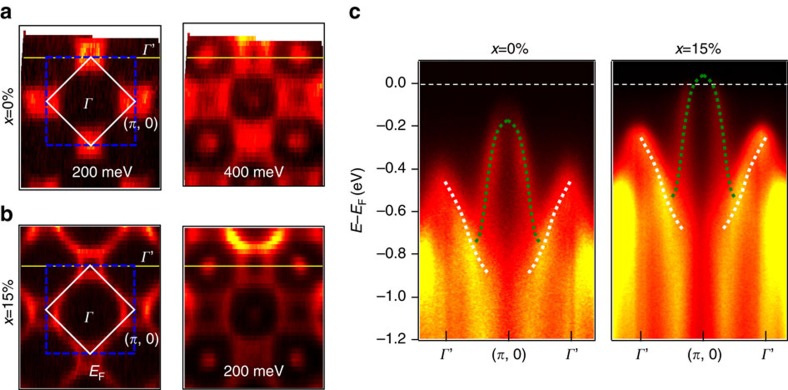
Constant energy surfaces and high-symmetry cuts of the parent and Rh-doped Sr_2_IrO_4_. (**a**,**b**) The constant energy surfaces of the parent (**a**) and *x*=15% Rh-substituted (**b**) Sr_2_IrO_4_. The numbers are binding energies relative to *E*_F_. The solid white/dashed blue lines are the folded/original BZs. (**c**) ARPES energy-momentum intensity plots along *Γ*'−(*π*, 0)−*Γ*' (yellow lines in panels (**a**,**b**)). The dashed green and white lines through the data guide the eye for the *J*_1/2_ and *J*_3/2_ bands, respectively.

Angle-resolved photoemission spectroscopy (ARPES) proves an invaluable tool for directly observing both electronic structure and low-energy electron dynamics in doped Mott insulators^3^ and bears implication for electronic order, including density-wave instabilities. We performed ARPES on single crystals of Rh-doped Sr_2_IrO_4_. Details of the sample growth are listed in the Methods section and Supplementary Note 1; and the ARPES experiment setups are presented in the Methods section and Supplementary Note 2. Due to the 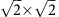 lattice reconstruction, the Brillion zone (BZ) of Sr_2_IrO_4_ and its Rh-doped compounds is reduced in half, similar to the formation of the antiferromagnetic (AF) BZ in the parent cuprates. We show the constant energy surfaces of Sr_2_IrO_4_ (Fig. 1b, left panel) and as a comparison, of Pb-doped Bi_2_Sr_2_CaCu_2_O_8+δ_ (Fig. 1b, right panel). The folded and ‘original' BZs for the iridates are marked in white solid and blue dashed lines, respectively. The ‘original' BZ corresponds to one Ir-O plaquette in real space, as shown by the blue dashed line in the left panel in Fig. 1a. As we will show later in the paper, the close-to-*E*_F_ features of the doped iridates are best captured not by the folded BZ, but instead by the ‘original' blue BZ. To avoid confusion, we use (*π*, 0) to mark the X point in the BZ as defined in ref. 9, and use *Γ*' to denote (*π*, *π*), which is the *Γ* point in the second folded BZ. In Fig. 2a,b, we show the constant energy surfaces as a function of binding energy for Sr_2_IrO_4_ and Sr_2_Ir_1−*x*_Rh_*x*_O_4_ with *x*=15% at T=50 K. While there is no Fermi surface for the parent compound, there are states at *E*_F_ in the *x*=15% iridate, corresponding to enhanced conductivity in the Ir-O plane. The constant energy surface of the *x*=15% compound is quite similar to that of the parent, except that it is shifted in binding energy by ∼200 meV. To identify the Fermi surface topology, we plot the ARPES spectrum along *Γ*'−(*π*, 0) for both samples (Fig. 2c; along the yellow lines in Fig. 2a,b). There is a hole-pocket centred at (*π*, 0), which comes from the *J*_1/2_ LHB^9^,^18^. The top of the valence band is ∼170 meV below *E*_F_ in the parent compound, and is above *E*_F_ for the *x*=15% sample. Indeed, both the *J*_3/2_ band (white dashed line in Fig. 2c) and J_1/2_ LHB (green dashed line)^19^,^20^ are shifted by ∼200 meV. It appears that rather than a reduced splitting of the *J*_3/2_ and *J*_1/2_ bands, Sr_2_IrO_4_ is hole doped with Rh substitution. Note, that while the Rh-doped compound displays strong spectral weight extending towards (*π*/2, *π*/2), the M point near *E*_F_, the band dispersion at the M point lies below *E*_F_ (see Supplementary Fig. 2 and Supplementary Note 3). Therefore, the ‘Fermi surface' is made up only of the states encircling X or (*π*, 0), that is, it encompasses holes.

**Figure 3:**
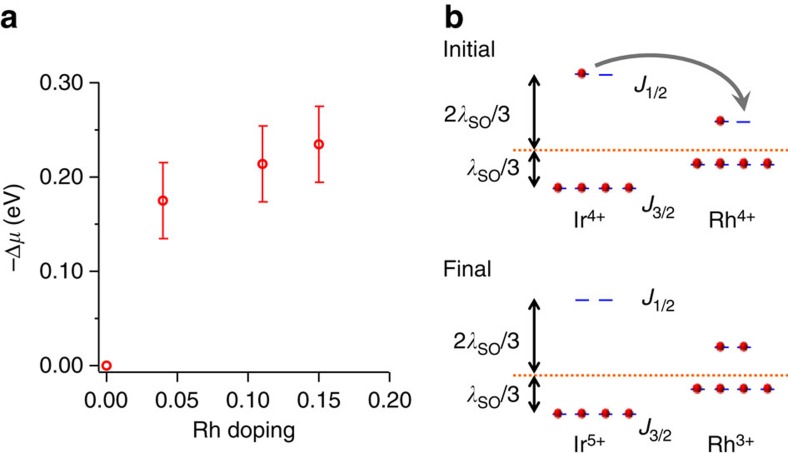
Rh atoms act as hole dopants in Sr_2_IrO_4_. (**a**) Chemical potential shift versus Rh concentration. The chemical potential shift is measured from the shift of both *J*_1/2_ LHB and *J*_3/2_ bands. The error bar for each Rh concentration is the half-width of the positive peaks of the second-derivatives of EDCs, averaged over a few high-symmetry k points. (**b**) Simple atomic picture of hole doping, ignoring band effects and Mott splitting. With a roughly similar average energy for both Ir and Rh sites, the smaller on-site spin-orbit splitting on the Rh sites lowers the *J*_1/2_ energy relative to that of the host Ir sites. This causes an electron transfer to Rh, that is, hole doping of the Ir lattice.

We determined the chemical potential shift quantitatively from the valence bands, as shown in Fig. 3a. With the increase of Rh concentration, the chemical potential is pushed deeper into the *J*_1/2_ LHB, confirming that Rh acts as an effective hole dopant. We extrapolate the chemical potential shift at finite Rh densities and derive an ∼170 meV intercept in the zero doping limit. Note, the top of the valence band in the parent compound locates at (*π*, 0) and is around 170 meV below *E*_F_, from both ARPES^18^ and scanning-tunneling^21^ experiments. Thus on effective hole doping the chemical potential immediately jumps to the edge of the LHB (Fig. 1d), as opposed to competing models (Fig. 1e)^16^,^22^ where new quasiparticle-like states emerge in the middle of the Hubbard gap. The doping schematic in Fig. 1d also agrees with the recent optical conductivity measurements^16^, while the interpretation in ref. 16 (Fig. 1e) could not explain the ARPES measured band structure (see Supplementary Note 4).

The effective hole doping is quite plausible when considering the simple atomic model depicted in Fig. 3b. Rh atoms have smaller spin-orbit coupling than Ir, leading to the smaller splitting between the *J*_3/2_ and *J*_1/2_ states. Assuming the average energy of all six t_2g_ states is similar for both Rh and Ir, the empty *J*_1/2_ state of Rh would then have a lower energy than that of Ir. Thus a *J*_1/2_ electron from a neighbouring Ir atom will fill the *J*_1/2_ state on the Rh site, leaving behind a hole on the Ir site, as well as a filled and immobile Rh^3+^ site. Of course this is a simplistic model that neither takes into account the finite bandwidth of the effective J states nor the Coulomb repulsion U. Recent X-ray absorption experiments at the Rh L_3_-edge have confirmed Rh indeed has a valence of 3+ in these compounds^23^.

### Fermi arcs and pseudogaps

**Figure 4:**
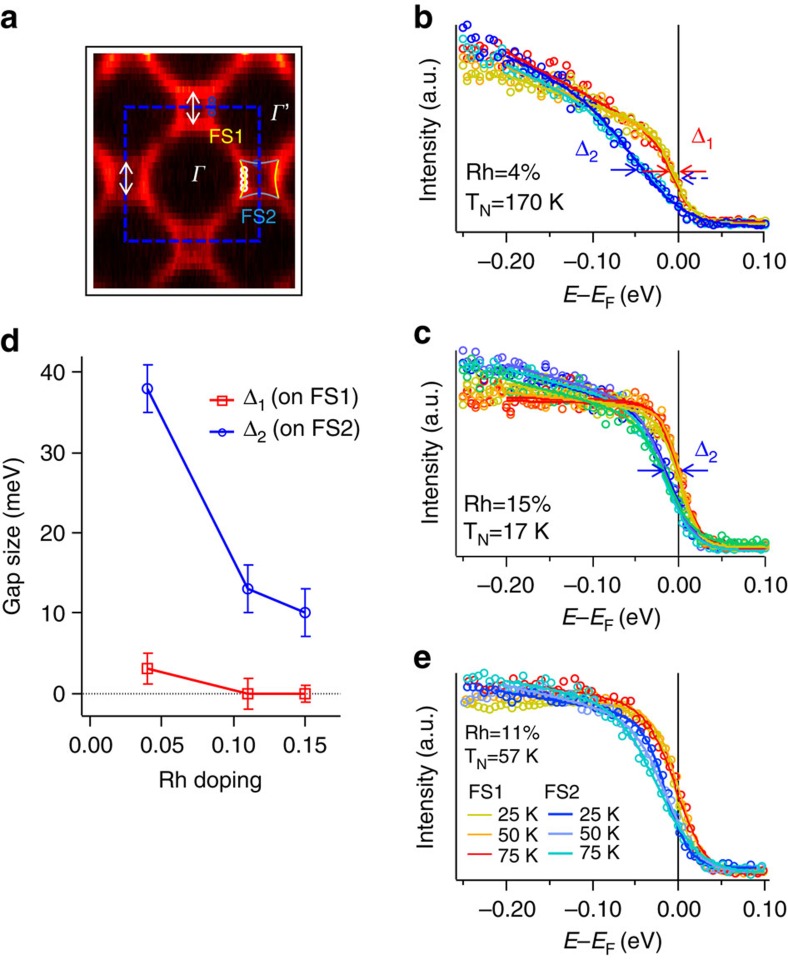
Fermi surface segments and pseudogaps in hole-doped Sr_2_IrO_4_. All the data were taken at 50 K unless otherwise noted. (**a**) Fermi surface spectral weight of the *x*=15% sample, with a hole-like Fermi pocket centred around the (*π*, 0) point of the unfolded (blue dashed) BZ. The FS pocket is separated into segments FS1 (yellow) and FS2 (blue), with FS1 facing *Γ* and FS2 facing *Γ*'. Q vectors (white arrows) are possible density-wave nesting vectors. (**b**,**c**) EDCs from multiple locations along the FS1 and FS2 segments (yellow and blue, respectively) taken from the *x*=4% and *x*=15% samples. Locations of the individual EDCs are marked by the open coloured circles in **a**. The leading edges of most EDCs do not reach E_F_, that is, they are gapped or pseudogapped. Gap sizes extracted using the ‘midpoint of leading edge' method, are shown in **b**,**c** and compiled in **d**, with Δ_1_ labelling the gaps from FS1 and Δ_2_ the gaps from FS2. The uncertainties of Δ_1_ and Δ_2_ are defined as the standard deviation of the fitted gap sizes of individual EDCs from the averaged value. (**e**) EDCs from FS1 (dashed) and FS2 (solid) showing minimal temperature dependence across the magnetic phase transition of the *x*=11% sample.

Hereafter, we focus on the low-energy electronic dynamics of these hole-doped compounds in search for ‘universal' features during the Mott-metal crossover. In Fig. 4a we show the Fermi surface topology for the *x*=15% sample at 50 K. The segments of the Fermi surface centred at *Γ* (*Γ*') and equivalent k locations are highlighted with solid yellow (blue) lines, and labelled FS1 and FS2, respectively. Energy-distribution curves (EDCs) from many different k points on the Fermi surface are plotted in Fig. 4c. There is a marked difference between the EDCs from FS1 and FS2—those from FS2 are generally pushed away from *E*_F_. We refer to this spectral weight suppression as a ‘pseudogap' and use the standard ‘midpoint of leading edge' method^24^,^25^ to quantify it, by fitting the EDCs to a shifted, broadened leading edge (see Supplementary Note 5), with the amount of shift (defined as the gap size) giving the 50% point of the leading edge. Note that in contrast to the case of a BCS-like gap that works well for superconductivity or a standard charge or spin density-wave gap, this shifted edge does not have a pile-up of spectral weight beyond the gap edge, like many other pseudogaps^4^,^25^, that is, it does not enforce spectral weight conservation on the opening of the gap. This absence of quasiparticle peaks in the doped iridates is one of the signatures of their non-Fermi liquid nature. We assign the gap size Δ_1_ and Δ_2_ from the leading edge fitting to FS1 and FS2, respectively. For *x*=15%, Δ_1_ as defined by this leading edge method vanishes, and FS1 can be considered a ‘regular' piece of Fermi surface (albeit without quasiparticle peaks), while EDCs from FS2 show a partial depletion of near-*E*_F_ spectral weight. As FS1 is only topologically connected to the ‘gapped' FS2, we describe the FS1 as a Fermi surface ‘arc' and FS2 as ‘pseudogapped'.

We track how Δ_1_ and Δ_2_ evolve with reduced Rh concentration, as the material gets closer to the Mott insulator. At *x*=4% (Fig. 4b), both Δ_1_ and Δ_2_ are finite, indicating that both FS1 and FS2 are pseudogapped, with Δ_1_∼3 meV and an increased Δ_2_∼38 meV. In Fig. 4d we plot the doping dependence of Δ_1_ and Δ_2_. For *x*=4%∼<11%, the entire Fermi surface is pseudogapped, which resembles the deeply underdoped cuprates^26^,^27^. We mark the presence of both pseudogaps as suppressed spectral weight near *E*_F_ (the ‘notch') in Fig. 1d. The pseudogaps observed here are not to be mistaken for the matrix element effect as these gaps happen over a very narrow-binding energy range that is essentially identical for different photon energies (see Supplementary Note 6).

The pseudogap phase in correlated electron systems is often considered a symmetry-broken phase of matter. Thus the origin of the pseudogap could be reflected in its k-space symmetry, as well as its thermal evolution. For Rh concentration 4–15%, Δ_1_ (Δ_2_) is roughly independent of **k** along the Fermi surface segment FS1 (FS2). In this sense, the pseudogaps in the non-superconducting Rh-doped iridates are clearly different from the near-nodal prepairing pseudogap in the near-optimal cuprates^28^, where pseudogaps follow the superconducting paring symmetry.

Figure 4e shows the temperature dependence of EDCs at k_F_ from FS1 and FS2 for the *x*=11% sample, with the temperature range straddling the AF ordering temperature T_N_=57 K. Within the error bar no obvious changes with temperature are observed, indicating that the pseudogap is not directly related to the long-range canted AF order. This is further confirmed in the *x*=15% sample (Fig. 4c), where Δ_2_ is finite at 50 K and persists to at least 200 K (see Supplementary Fig. 3 and Supplementary Note 5), above T_N_=17 K. This observation suggests the pseudogap is not tied to the long-range magnetic order, and it is likely the pseudogap phase persists down to the zero-temperature quantum ground state in these hole-doped iridates.

Another commonly considered origin of pseudogaps is the density-wave instabilities in the form of Fermi surface nesting, as has been discussed in the manganites^25^ and cuprates^6^,^29^. In the case of iridates, it is temping to draw nesting vectors such as the white arrows (Fig. 4a) between FS2's with the same gap size Δ_2_. However, the same ordering vector Q also connects FS1's, yet with a much smaller gap Δ_1_. The Fermi surface nesting scenario does not explain the preference for a larger gap along FS2 than along FS1. It appears the pseudogap in iridates is inconsistent with many influential explanations for the antinodal pseudogap^25^,^29^ in manganites and near optimally doped cuprates though it may be more connected with the more-recently observed nodal pseudogap in heavily underdoped cuprates^26^,^27^.

### Marginal-Fermi-liquid like single-electron scattering rate

**Figure 5:**
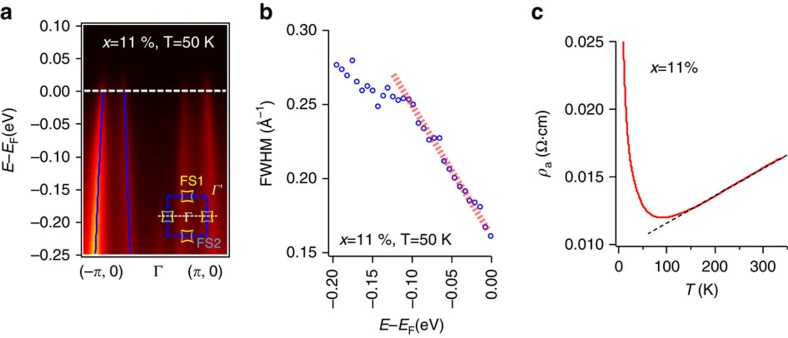
Marginal-Fermi-liquid-like single-electron scattering rates. (**a**) The energy-momentum intensity plot along (−*π*, 0)−*Γ*−(*π*, 0) for an *x*=11% sample at T=50 K, passing through four pieces of FS1 Fermi surface. The peak centroids obtained from double-Lorentzian fittings to the momentum-distribution curves (MDCs) are marked with blue lines. The inset shows where the cut in the main figure is taken from the BZ. (**b**) The full-width-half-maximum (FWHM) from the double-Lorentzian fitting is plotted versus the binding energy, showing a linear ‘MFL' scattering rate. (**c**) Resistivity versus temperature for the same sample showing a linear dependence at intermediate temperatures. The black dashed line is a guide to the eye.

The non-Fermi liquid nature of these doped iridates is not only reflected in the absence of quasiparticle peaks along the EDC (Fig. 4b,c), but also in the single-electron scattering rate. In the Fermi liquid theory, the quasiparticle scattering rate grows linearly with the binding energy (and temperature) squared. For up to 15% Rh substitution, as shown in Fig. 5b (with raw data shown in Fig. 5a) the scattering rates increase roughly linearly with binding energy—a signature of the MFL^5^. We could rule out this linear scattering rate as from the Rh-Ir substitution, and further details are shown in the Supplementary Fig. 4 and Supplementary Note 7. Moreover, as shown in Fig. 5c, there is a linear relation between the resistivity and the sample temperature, as highlighted by the black dashed line. Here we have ignored the upturn of the resistivity at low temperatures that is likely due to a localization effect, as has also been observed in most of the underdoped cuprates^30^. The linear MFL scattering rate is one of the most iconic features of the cuprates and other correlated materials such as the ruthenates^31^ and has been attributed to a wide range of ideas including quantum critical fluctuations^5^ and the break-up of quasparticles^32^.

## Discussion

The simultaneous appearance of Fermi arcs, pseudogaps and the MFL state in both iridates and cuprates is unlikely to be coincidental. We note recent measurements of electron-doped iridates using a range of spectroscopies, including ARPES^33^,^34^,^35^, scanning-tunneling microscope^36^ and resonant inelastic X-ray scattering^37^, also with features similar to those in cuprates. Our study and these new findings suggest there is a wide resemblance between doped iridates and cuprates in terms of band structures, electron dynamics and collective excitations, and on both the hole and electron-doped sides of the phase diagram. As cuprate is a canonical doped Mott insulator, and the most thoroughly studied one so far, our knowledge of a doped Mott insulator is to a great extend bounded by the cuprate literature. In this sense, the aforementioned hallmarks are most likely intrinsic and universal, rather than material specific, during the Mott-metal crossover.

We have ruled out prepairing and Fermi surface nesting as accountable for the pseudogaps in the iridates. Also there is no known quantum critical point in the doped iridates. We could also rule out long-range AF and structural distortions: both the pseudogap and the MFL survive above T_N_ in the iridates, and static structural distortions are less likely to be related to the electron dynamics.

The precise definition of a pseudogap is in itself debated. From an experimental perspective, a widely accepted working criterion is well-articulated by Timusk and Statt^4^ in the mid-1990s in the study of cuprates: a ‘pseudogap' is a partial gap, or more general, an incomplete suppression of spectral weight, that occurs only on some regions of the Fermi surface. Experimental features observed in, for example, ARPES, scanning-tunneling microscope, optical conductivity, all stem out of this seemingly naive (and more general) working definition. Admittedly, this definition does not point to the nature of pseudogaps. There are some cases where the origin of pseudogaps are understood theoretically to various degrees. For example, pseudogaps from preformed Cooper pairs usually have a momentum dependence characteristic of the pairing symmetry^3^,^28^,^38^, with relatively well-defined Bogoliubov quasiparticles. On the other hand, pseudogaps due to Fermi surface nesting features ‘hotspots' on the Fermi surface connected by the charge/orbital/magnetic ordering vector^25^,^29^, which could be detected in other techniques.

Notably, even in these cases, not all facets of the pseudogap of interest, for example, its evolution against temperature, follows known theories. For example, the prepairing pseudogap^28^ in the optimally doped cuprates does evolve continuously into a ‘real' hardened gap with decreasing temperature into the superconducting regime. However, as temperature rises, the prepairing pseudogap has been reported^38^,^39^ to be killed due to density of states filling-in rather than a reduction in gap size with increasing temperature, unlike standard Bogoliubov quasiparticles, reflecting their non-Fermi liquid nature. In other cases, a ‘real' hardened gap and a defined phase transition may never develop when lowering the temperature towards absolute zero from the pseudogap phase. For example, in the p-type cuprates with a hole doping of 5–6%, the pseudogap temperature is generally above 200 K, and the ground state is considered by many a spin-glass without any defined phase transition^3^,^40^. There is no known clear boundary between the pseudogap phase and the spin-glass in this regime.

As we pointed out at the forefront of this paper, here we define pseudogaps following the definition from Timusk and Statt^4^. We have shown the pseudogaps and Fermi arcs in these iridates are distinctive from the prepairing and charge/orbital/spin ordered ones. The survival of pseudogaps to these seemingly high temperatures, compared with the Neel temperature, is a reflection of the Mott nature of iridates—something that is very relevant considering the recent debates over whether Sr_2_IrO_4_ is a Mott insulator or a Slater insulator^41^,^42^. The main disagreement between the two types of insulators is the role of short-range AF correlations. The Slater insulator is a mean-field concept built on the formation of long-range order—in the present case the long-range AF order. The short-range AF correlations that underlie the long-range AF order is ignored, or at best considered as a higher-order fluctuation. It is expected that the gap in a Slater insulator will diminish with decreasing long-range magnetic order, trending to zero as the phase transition is approached. Experimentally, there is no clear change in the band structure for both the parent compound^18^,^43^ and the doped iridates (this work) across the onset of long-range magnetism. This indicates the long-range magnetic order is not necessary for the formation of the gap and Sr_2_IrO_4_ is a Mott insulator.

Our observation of hallmarks during the Mott-metal crossover due to their proximity to the Mott state is in contrast with many of the prevailing views in the literature, but similar to those suggested in a few recent theoretical works^44^,^45^. More specifically, the smooth crossover from the Mott insulator Sr_2_IrO_4_ to the hole-doped ‘bad metal', suggests the essential role of short-range correlations in the low-energy electron dynamics. It is interesting to point out the short-range AF correlations may be responsible for many of the hallmarks during the Mott-metal crossover, and in regions of the phase diagram where long-range AF order is long gone. For example, short-range AF correlations could indicate a widespread distribution of AF interaction strengths and quantum incoherence, giving rise to the lack of quasiparticle peaks and less well-defined gapping near the Fermi level—a possibility for iridates and even cuprates, among other doped Mott insulators.

The relation between the AF energy scale and the pseudogap temperatures is far from clear, with the strengths of the AF interactions converted to >1,000 K in iridates (and cuprates). In the latter the pseudogap temperature T* neither tracks the AF phase nor the superconducting dome. As for iridates, at this stage, we are not yet sure whether the pseudogap closes or could be filled due to thermal effects, at a higher temperature T*. Our data here would suggest most likely that there is a gradual filling-in of the density of states with rising temperature, while the actual T* (if it can be well-defined) is yet to be determined (see Supplementary Note 5 for details). Interestingly the community does not have a consensus whether the pseudogap actually closes or is filled in the underdoped cuprates. Regardless, this type of study, in which we closely consider the similarities and differences in the behaviour of the iridates and cuprates as they evolve away from the Mott insulator is a powerful tool to delve deeply into the nature of the doped Mott insulating state.

## Methods

### Sample preparation

All the ARPES, transport and magnetization data were taken from bulk Sr_2_Ir_1−*x*_Rh_*x*_O_4_ samples. Single crystals were grown from off-stoichiometric quantities of SrCl_2_, SrCO_3_, IrO_2_ and RhO_2_ using self-flux techniques.

### ARPES measurements

The ARPES experiments were performed at the PGM-A end station at the Synchrotron Radiation Center of University of Wisconsin-Madison, the Beamline 4.0.3 and 7.0.1 ARPES end stations at the Advanced Light Source, Lawrence Berkeley National Laboratory and the Surface and Interface Science beamline of the Swiss Light Source at the Paul Scherrer Institut. The samples were cleaved *in situ* with vacuum better than 5 × 10^−11^ Torr. The band structure and low-energy spectra near the Fermi level were taken with *hν*=77, 80, and 90 eV, with an energy resolution ∼25 meV, which is sharp on the scale of the principle spectral features.

## Additional information

**How to cite this article:** Cao, Y. *et al.* Hallmarks of the Mott-metal crossover in the hole-doped pseudospin-1/2 Mott insulator Sr_2_IrO_4_. *Nat. Commun.* 7:11367 doi: 10.1038/ncomms11367 (2016).

## Supplementary Material

Supplementary Information Supplementary Figures 1-4, Supplementary Notes 1-7 and Supplementary References
