# Supplementary material for: Hallmarks of the Mott-metal crossover in the hole-doped pseudospin-1/2 Mott insulator Sr2IrO4
Source: Nat Commun. 2016 Apr 22;7:11367. doi: 10.1038/ncomms11367 (PMC4844699; doi:10.1038/ncomms11367)
Supplement: Supplementary Information — Supplementary Figures 1-4, Supplementary Notes 1-7 and Supplementary References [file ncomms11367-s1.pdf]

Supplementary Information

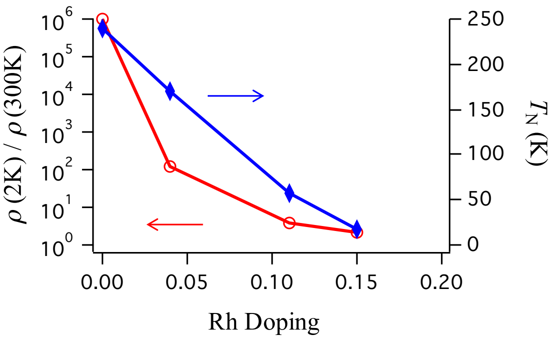

Supplementary Figure 1. The evolution of resistivity and magnetism with Rh doping. The left axis: the normalized resistivity; and the right axis the onset of the long-range magnetic order.

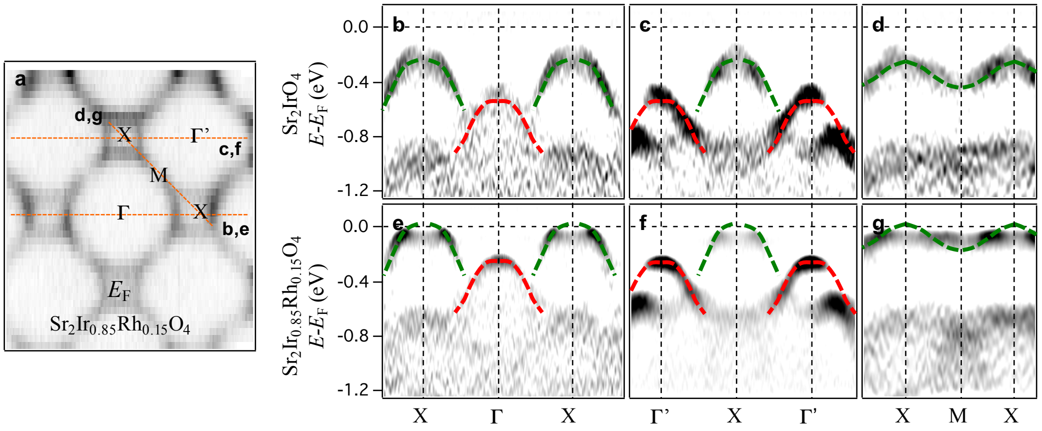

Supplementary Figure 2. The band structure along high symmetry directions in the parent and Rh doped Sr_2IrO_4. a. The Fermi surface topology of the x=15% Rh-doped sample. The high symmetry cuts in panels **b** to **g** are drawn in dashed orange line. **b-g.** The EDC second-derivative maps for the parent (**b-d**) and x=15% Rh-doped sample (**e-g**)

respectively. The dashed lines are guides to the eye where the green and red colors denote the $J_{1/2}$ lower Hubbard band and $J_{3/2}$ band.

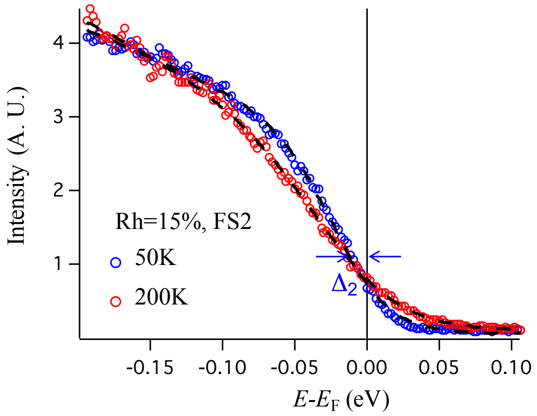

Supplementary Figure 3. The temperature dependence of the pseudogap Δ_2. The EDCs from the gapped FS2 of an x=15% sample, taken at 50K and 200K respectively. The black dashes are fits using the "midpoint of leading edge" method as described in the Supplementary Equation (1).

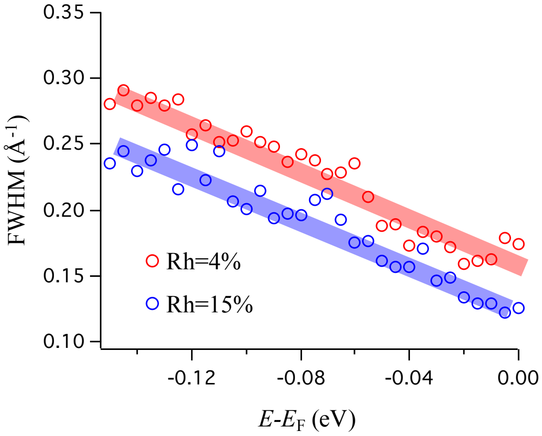

Supplementary Figure 4. Comparison of MDC widths (single-electron scattering rates) as a function of doping, along FS1 of the Fermi surface. The more heavily doped samples have smaller scattering rates.

Supplementary Note 1

Bulk crystal growth and characterization

All the transport, magnetization and photoemission experiments were performed on bulk $Sr_2Ir_{1-x}Rh_xO_4$ single crystalline samples grown from off-stoichiometric quantities of $SrCl_2$, $SrCO_3$, IrO_2 and RhO_2 using self-flux techniques. These mixtures were heated to 1480°C in Pt crucibles, fired for 20 hours, cooled at 5°C/hour to 1440°C, and then rapidly quenched to room temperature. The quenching process is critical to ensure an exclusion of precursor phases such as Sr_2IrO_4.

In Supplementary Figure 1, we show the normalized resistivity (left axis) and the onset temperature of the long-range magnetic order (right axis) as a function of Rh concentration. The long-range antiferromagnetism manifests itself as a weak ferromagnetism in the magnetic susceptibility, due to the canted Ir moment in the Ir-O plane. This is also why we call the onset temperature as the Neel temperature.

It is interesting to note that the insulating behavior melts rapidly (as the resistivity is drawn using log scale), while the long-range magnetic order did not vanish till 15%. The resistivity/magnetism phase diagram in the Rh doped Sr_2IrO_4 shares intriguing similarity to the phase diagram, e.g. of $(La_{1-x}Sr_x)_2CuO_4$ [1]. Further information, including the transport and magnetization measurements are described in Ref. [2].

Supplementary Note 2

ARPES Experiment Setup

The ARPES (angle-resolved photoemission spectroscopy) experiments were performed at the PGM-A endstation at the Synchrotron Radiation Center (SRC) of University of Wisconsin-Madison, the Beamline 4.0.3 and 7.0.1 ARPES endstations at the Advanced Light Source, Lawrence Berkeley National Laboratory and the Surface and Interface Science (SIS) beamline of the Swiss Light Source at the Paul Scherrer Institut. The samples were cleaved *in situ* with vacuum better than 5×10^{-11} Torr. The band structure

and low-energy spectra near the Fermi level were taken with $h\nu$=77eV, 80eV, and 90eV, with an energy resolution ~25meV.

Supplementary Note 3

Band structure of $Sr_2Ir_{1-x}Rh_xO_4$

We compare the band structures of the Mott insulator Sr_2IrO_4 and of the x=15% Rh-substituted sample. Due to the local rotation of the Ir-O octahedra, there is a $\sqrt{2} \times \sqrt{2}$ lattice reconstruction in the whole temperature range where ARPES experiments are performed (and in fact up to the room temperature). The Brillouin zone (BZ) is folded for both compounds. To compare to existing first-principles and dynamical mean-field calculations, (π, 0) point corresponds to the X point and (π/2, π/2) the M point. As shown in the main text, while the folded BZ suffices to describe the band dispersion, the "original" BZ corresponding to one Ir-O plaquette most closely captures the low-energy electron dynamics. For this reason we define (π, π) as the Γ' point, to distinguish it from the Γ point at (0, 0) or (2π, 0).

To find the band structure we may track the peaks as shown in Figure 2 of the main paper or we may take the second derivative of the energy distribution curves (EDCs) as shown in Supplementary Figure 2. This technique is routinely used to highlight the band dispersion in ARPES experiments [3, 4, 5]. We obtain the band structures from the positive peaks on these EDC second-derivative maps, and by comparing band dispersions in the folded and "original" BZs. For example, for cuts along X-Γ-X and Γ'-X-Γ', while they are not equivalent in terms of the low-energy dynamics, e.g. pseudogaps, they have the same large-energy-scale band dispersion. For both the parent compound and the x=15% sample, the $J_{1/2}$ lower Hubbard band (LHB) and $J_{3/2}$ band are drawn in dashed green and red lines respectively. The J character of the valence bands is assigned as in [6, 7]. It is evident that both the $J_{1/2}$ LHB and $J_{3/2}$ bands moved towards E_F, with neither major relative shift between these two bands nor bandwidth renormalization.

A conservative estimate of the uncertainty of this procedure in determining the peak positions is the half-width of the positive peaks of the second-derivative curves. The

chemical potential shift is defined as the average shift in energy by comparing the band structures of the parent compound and the Rh substituted samples. The uncertainty of the chemical potential shift is then the sum of the uncertainties of the peak positions of both the pristine and the doped compounds. For Figure 3a in the main text we have determined these uncertainties at a few high symmetry k points and averaged their results.

Supplementary Note 4

Comparison to optical conductivity measurements

In Figure 1d and 1e of the main text we present two possible scenarios how Rh substitution bring metallicity into the parent compound. Figure 1e is similar to the proposal in Ref. [8]. In Figure 1d, the chemical potential "jumps" immediately to the lower Hubbard band with hole doping, while in Figure 1e there is in-gap spectral weight transferred from the upper and lower Hubbard bands and the chemical potential is pinned to this in-gap spectral weight.

The optical conductivity experiments could not distinguish between these two scenarios. The α and β transition peaks seen in the optical conductivity [8] are interpreted as the optical transition from the $J_{1/2}$ lower Hubbard band and $J_{3/2}$ band to the $J_{1/2}$ upper Hubbard band, respectively. For the Rh concentrations less than 20%, the α and β peak locations do not change – an indication there is not much relative shift between the $J_{3/2}$ band and $J_{1/2}$ Hubbard bands. This is consistent with both scenarios in Figure 1d and Figure 1e. The ARPES experiment as presented here, however, unambiguously shows that there is a sudden jump in the chemical potential. Moreover, the low energy peak that appears below the α peak in the Rh substituted samples could also be explained in Figure 1d. Since there is suppression of spectral weight near E_F for the Rh doped samples, the low energy peak could originate from the intra-band optical transition across the pseudogap, without introducing in-gap states near the middle of the gap as in Figure 1e.

Supplementary Note 5

Quantifying the pseudogap

As discussed in the main text, the EDCs do not have sharp quasiparticle peaks near the Fermi level. Similar broad EDCs have also been observed in lightly doped cuprates [9] as well as manganites [10]. This indicates the effectively hole-doped iridates have large scattering rates, consistent with the non-Fermi Liquid linear scattering rates shown in Figure 5 of the main text. To characterize the gap, we use the "midpoint of leading edge" method and fit the EDCs to (with ω being the energy relative to E_F):

$$I(\omega) = \frac{A+B\omega}{1+e^{(\omega+\Delta)/k_B T^*}} \quad (1)$$

which is essentially a Fermi function with variable edge width $k_B T^*$ and with the leading edge midpoint shifted from the chemical potential by the pseudogap value Δ. A, B are fitting coefficients describing a very simplistic form of spectral function. An example fit for two different temperatures is shown in Supplementary Figure 3.

For each Rh doping, the pseudogap sizes Δ_1 and Δ_2 are taken to be the average value of more than 10 EDCs on the Fermi surface, along FS1 and FS2 respectively. Accordingly, the uncertainties of Δ_1 and Δ_2 shown in Figure 4d are defined as the standard deviation of the fitted gap size of individual EDCs from the averaged value.

In Supplementary Figure 3 we stack the EDCs from FS2 in the x=15% sample at 50K and 200K. Clearly Δ_2 survives to more than 10 times the Neel temperature (17K) for this doping level, with more of a "filling in" rather than "gap closing" behavior as temperature is increased. While a related pseudogap in cuprates is typically discussed as closing to zero at the high temperature T* in cuprates, we note that a second look at many classic measurements of the temperature dependence of the pseudogap [11, 12, 13] actually indicate that the gap evolution is better described as a filling rather than closing behavior, consistent with the present findings on the iridates. Whether the gap in the iridates fully disappears (by either filling or closing) at a much higher temperature than the 200K will take much further experimentation (In the experiments performed here we refrained from going above 200K because of concerns of compromised vacuum conditions/surface ageing at these higher temperatures).

Supplementary Note 6

Spectral weight suppression separated from possible matrix element effects

In this section we justify that the low-energy spectral weight suppression (pseudogap) in the Rh doped Sr_2IrO_4 is not the result of the "matrix element effect". By "matrix element" [14], we refer to the electron-photon cross-section $I \propto \left|\langle\psi_f|\boldsymbol{A}\cdot\boldsymbol{p}|\psi_i\rangle\right|^2 \propto |\langle\psi_f|\boldsymbol{E}\cdot\boldsymbol{r}|\psi_i\rangle|^2$. Here I is the measured ARPES intensity, $\boldsymbol{A}$ is the electromagnetic gauge and $\boldsymbol{p}$ is the electron momentum. $|\psi_i\rangle$ and $|\psi_f\rangle$ are the wavefunctions of the initial state of the electron in the solid and the final state of the photo-excited electron, respectively. For certain combinations of the initial state, final state, and photon dipole operator it is in general possible that the matrix element becomes very small or vanishes, giving small ARPES spectral weight. It is important to be able to confirm that the pseudogaps observed in the iridates are not due to such matrix element effects.

We have measured the doped iridates in up to 8 inequivalent sectors of FS1 and FS2, have used two different sample rotations ((π,0) parallel to the scattering plane and (π,π) parallel to the scattering plane), and multiple photon energies (77eV, 80eV and 90eV). In all cases we have observed the same qualitative behavior of a large pseudogap along FS2 and a weak or small pseudogap along FS1. While strongly varying matrix elements might have affected the data at one geometry or photon energy, it is not plausible that they could have affected the data for all experimental conditions we have explored.

Moreover, the spectral weight is suppressed within <50meV of E_F across the whole Rh doping range for both FS1 and FS2. Spectral weight suppression originating from the initial-state orbital selectivity ("matrix element effect") usually appears over a much larger energy scale, which is not consistent with the observed pseudogap size in the hole-doped iridates.

Supplementary Note 7

The role of Rh atoms in the low-energy electron dynamics

We argue that the pseudogap does not directly arise due to disorder effects from the alien Rh atoms in the Ir-O plane, though there may be second order connections. This is based on the following key observations:

First, the sizes of the gaps (both Δ_1 and Δ_2) shrink with the increase of Rh concentration. Additionally, the single-electron scattering rate obtained from MDC analysis is smaller in the 15% sample than in the 4% sample (Supplementary Figure 4). These are consistent with increased conductivity from Rh=4% to 15%, while backwards from the increase of the number of alien scattering centers in the doped iridates.

Second, there is no clear theoretical link between disorder and pseudogap / marginal Fermi liquid scattering rate. In fact, a study of highly-disordered graphene [15] suggested the near-E_F electron dynamics in the Anderson localization limit is very different from those reported in this work. Regarding the pseudogap, both in Anderson's original theory and in Ref. [15], there is no depletion of spectral weight near E_F. In terms of the electron scattering rate, Ref. [15] showed a pronounced broadening of the MDC width almost independent of the electron binding energy as the system enters the disordered regime. In the most disordered case, the MDC width is almost constant on the order of eV. Thus the linear-in-energy and linear in T marginal Fermi liquid behavior is unlikely a direct result of scattering off local disorders.

Supplementary References

[1] Ando, Y. *et al.* Mobility of the Doped Holes and the Antiferromagnetic Correlations in Underdoped High- T_C Cuprates. *Phys. Rev. Lett.* **87**, 017001 (2001).

[2] Qi, T. F. *et al.* Spin-orbit tuned metal-insulator transitions in single-crystal $Sr_2Ir_{1-x}Rh_xO_4$ ($0\leq x\leq 1$). *Phys. Rev. B* **86**, 125105 (2012).

[3] Sato, T. *et al.* Observation of dx^2-y^2-like superconducting gap in an electron-doped high-temperature superconductor. *Science* **291**, 1517-1519 (2001).

[4] Ronning, F. *et al*. Evolution of a metal to insulator transition in $Ca_{2-x}Na_xCuO_2Cl_2$ as seen by angle-resolved photoemission. *Phys. Rev. B* **67**, 165101 (2003).

[5] Yang, H.-B. *et al*. Fermi Surface Evolution and Luttinger Theorem inNa_xCoO_2: A Systematic Photoemission Study. *Phys. Rev. Lett.* **95**, 146401 (2005).

[6] Zhang, H., Haule, K., & Vanderbilt, D. Effective J=1/2 Insulating State in Ruddlesden-Popper Iridates: An LDA+DMFT Study. *Phys. Rev. Lett.* **111**, 246402 (2013).

[7] Martins, C., Aichhorn, M., Vaugier, L. & Biermann, S. Reduced Effective Spin-Orbital Degeneracy and Spin-Orbital Ordering in Paramagnetic Transition-Metal Oxides: Sr_2IrO_4 versus Sr_2RhO_4. *Phys. Rev. Lett.* **107**, 266404 (2011).

[8] Lee, J. S. *et al*. Insulator-metal transition driven by change of doping and spin-orbit interaction in Sr_2IrO_4. *Phys. Rev. B* 85, 035101 (2012).

[9] Yoshida, T. *et al*. Metallic Behavior of Lightly Doped $La_{2-x}Sr_xCuO_4$ with a Fermi Surface Forming an Arc. *Phys. Rev. Lett.* **91**, 027001 (2003).

[10] Chuang, Y.-D. *et al*. Fermi Surface Nesting and Nanoscale Fluctuating Charge/Orbital Ordering in Colossal Magnetoresistive Oxides. *Science* **292**, 1509-1513 (2001).

[11] Kugler, M. *et al*. Scanning tunneling spectroscopy of $Bi_2Sr_2CuO_{6+\delta}$: new evidence for the common origin of the pseudogap and superconductivity. *Phys. Rev. Lett.* **86**, 4911-4914 (2001).

[12] Timusk, T. & Statt, B. The pseudogap in high-temperature superconductors: an experimental survey. *Rep. Prog. Phys.* **62,** 61-122 (1999).

[13] Reber, T. J. *et al*. The origin and non-quasiparticle nature of Fermi arcs in $Bi_2Sr_2CaCu_2O_{8+\delta}$. *Nature Physics* **8**, 606-610 (2012).

[14] Damascelli, A., Hussain, Z. & Shen, Z.-X. Angle-resolved photoemission studies of the cuprate superconductors. *Rev. Mod. Phys.* **75**, 473-541 (2003).

[15] Bostwick, A. *et al*. Quasiparticle transformation during a metal-insulator transition in graphene. *Phys. Rev. Lett.* **103**, 056404 (2009).
